# The E3 ubiquitin ligase Hace1 is required for early embryonic development in *Xenopus laevis*

**DOI:** 10.1186/s12861-016-0132-y

**Published:** 2016-09-21

**Authors:** Akira Iimura, Fuhito Yamazaki, Toshiyasu Suzuki, Tatsuya Endo, Eisuke Nishida, Morioh Kusakabe

**Affiliations:** Department of Cell and Developmental Biology, Graduate School of Biostudies, Kyoto University, Sakyo-ku, Kyoto 606-8502 Japan

**Keywords:** HACE1, Early development, *Xenopus laevis*

## Abstract

**Background:**

HECT domain and ankyrin repeat containing E3 ubiquitin protein ligase 1 (HACE1) regulates a wide variety of cellular processes. It has been shown that one of the targets of HACE1 is the GTP-bound form of the small GTPase Rac1. However, the role of HACE1 in early development remains unknown.

**Results:**

In situ hybridization revealed that *Xenopus laevis hace1* is specifically expressed in the ectoderm at the blastula and gastrula stages and in the epidermis, branchial arch, kidney, and central nervous system at the tailbud stage. Knockdown of *hace1* in *Xenopus laevis* embryos via antisense morpholino oligonucleotides led to defects in body axis elongation, pigment formation, and eye formation at the tadpole stage. Experiments with Keller sandwich explants showed that *hace1* knockdown inhibited convergent extension, a morphogenetic movement known to be crucial for body axis elongation. In addition, time lapse imaging of whole embryos during the neurula stage indicated that *hace1* knockdown also delayed neural tube closure. The defects caused by *hace1* knockdown were partly rescued by knockdown of *rac1*. Moreover, embryos expressing a constitutively active form of Rac1 displayed phenotypes similar to those of embryos with *hace1* knocked down.

**Conclusions:**

Our results suggest that *Xenopus laevis hace1* plays an important role in early embryonic development, possibly via regulation of Rac1 activity.

**Electronic supplementary material:**

The online version of this article (doi:10.1186/s12861-016-0132-y) contains supplementary material, which is available to authorized users.

## Background

*HECT domain and ankyrin repeat containing E3 ubiquitin protein ligase 1* (*HACE1*) was identified as a gene located in an affected region of chromosome 6q21 in human Wilms’ tumor [1]. It has been shown that *HACE1* expression is reduced in multiple human tumors, and forced expression of *HACE1* in human tumor cell lines inhibits cell growth in vitro and in vivo [2]. Moreover, *Hace1*^−/−^ mice develop spontaneous late-onset cancer [2]. Therefore, *HACE1* is considered to be a tumor suppressor gene. HACE1 protein is a HECT family E3 ligase and is known to regulate multiple cellular processes. The most investigated target of HACE1 is Rac1. HACE1 specifically recognizes GTP-bound Rac1, an active form of Rac1, and promotes its degradation through the ubiquitin-proteasome pathway [3]. Rac1 degradation by HACE1 is important in regulating cell migration and reactive oxygen species (ROS) production [4, 5]. In addition, Rab GTPases are binding partners of HACE1 [6]. HACE1 regulates Golgi membrane dynamics during the cell cycle by interacting with Rab proteins [6]. One Rab GTPase Rab11a is activated by HACE1-mediated ubiquitination and subsequently promotes recycling of the β_2_-adrenergic receptor [7]. In addition, HACE1 has protective actions against oxidative stress in the brain and hemodynamic stress in the heart [8, 9]. Thus, these findings have demonstrated *HACE1* functions in diverse cellular processes. However, the roles of HACE1 during vertebrate embryonic development have not been previously reported in any model organism.

Here, we show that *Xenopus laevis hace1* is mainly expressed in the ectoderm and plays an important role in embryonic development. Hace1-depleted *Xenopus laevis* embryos displayed defects in multiple developmental processes. Analysis with Keller sandwich explants revealed that *Xenopus laevis hace1* is essential for convergent extension.

## Results

### *Xenopus laevis hace1* is mainly expressed in the ectoderm during early embryogenesis

To investigate a role of HACE1 in early embryonic development, we first cloned a *Xenopus laevis* HACE1 homolog (accession number AB894419) with a 2739 bp open reading frame. Compared with the reported sequence of *Xenopus laevis hace1* in GenBank (accession number NM_001093608), the *Xenopus laevis hace1* sequence that we obtained had five silent point mutations and a 96 bp deletion. These silent mutations may be single nucleotide polymorphisms. The 96 bp deletion may be caused by alternative splicing, because the human *HACE1* gene deposited in GenBank (accession number NM_020771) has a similar deletion. We thus concluded that the obtained *Xenopus laevis hace1* clone is a splicing variant. Human HACE1 protein shares 90 % and 87 % amino acid identity with our *Xenopus laevis* Hace1 protein and the previously reported protein, respectively. Hence, we used our clone in this study.

**Fig. 1:**
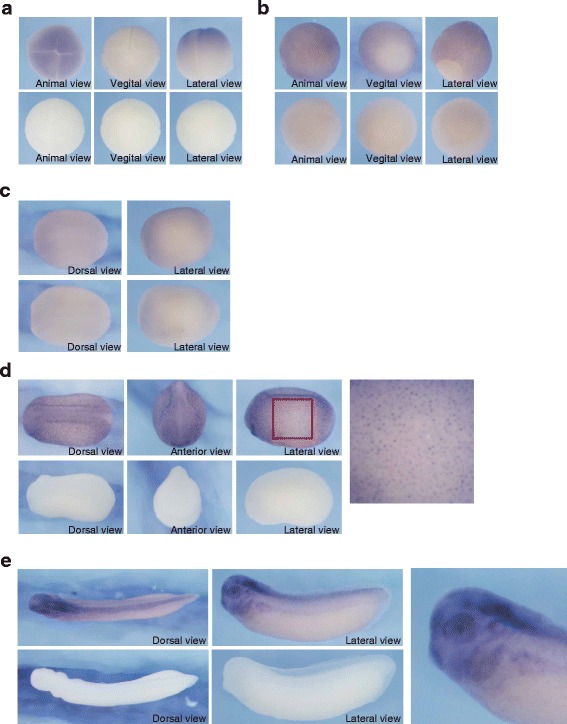
Spatial expression of *Xenopus laevis hace1* in early development. Expression of *hace1* during *Xenopus laevis* development was analyzed by whole-mount in situ hybridization using an antisense probe (upper panels) and sense probe as a control (lower panels). **a**, **b** At stage 4 (**a**) and 11 (**b**), *hace1* was expressed throughout the presumptive ectoderm. **c** At stage 15, *hace1* expression was weakly detected at the dorsal ectoderm. **d** At stage 23, *hace1* was expressed in the neural tissue and also showed spotty expression in the epidermis. The rightmost panel shows a magnification of the red boxed region. **e** *hace1* was expressed in the brain, eye, neural tube, branchial arch, ear vesicle, and kidney at stage 31. The right panel shows a magnified view of the embryo. Animal is to the top in lateral views (**a**, **b**). Dorsal is to the top in lateral or anterior views (**c**, **d**, **e**). Anterior is to the left in dorsal or lateral views (**c**, **d**, **e**)

Whole-mount in situ hybridization was performed to determine the expression pattern of *Xenopus laevis hace1*. Maternal transcripts of *hace1* were localized to the animal region (Fig. 1a). The expression was high in the presumptive ectoderm at the gastrula stage (Fig. 1b). At the neurula stage, *hace1* was weakly expressed in the dorsal ectoderm (Fig. 1c). At the early tailbud stage, *hace1* showed a spotty expression pattern in the epidermis and a uniform expression pattern in the neural tube (Fig. 1d). At the late tailbud stage, *hace1* was expressed in the brain, eye, neural tube, branchial arch, ear vesicle, and kidney (Fig. 1e).

### Knockdown of *hace1* causes shortening of the body axis and inhibition of eye and pigment formation

**Fig. 2:**
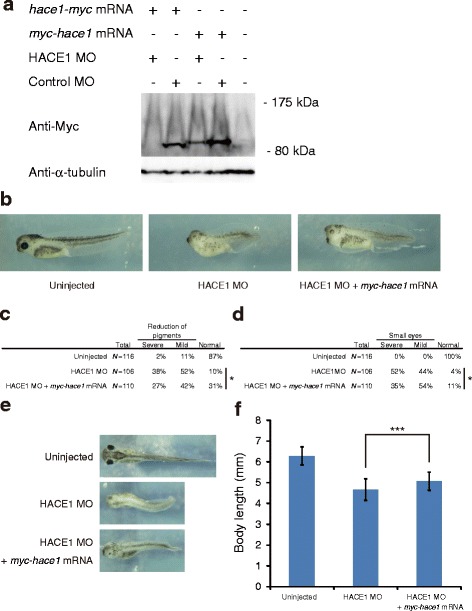
Dorsal depletion of *hace1* leads to multiple developmental defects. **a** Immunoblot analysis shows that HACE1 MO inhibited translation of *hace1-myc* mRNA but not that of *myc-hace1* mRNA used for rescue experiments. *Xenopus laevis* embryos were injected with the indicated sets of MOs (30 ng) and mRNAs (1.2 ng). α-tubulin was a loading control. **b** Embryos were injected with 30 ng of HACE1 MO alone (middle panel) or 30 ng of HACE1 MO plus 1.2 ng of *myc-hace1* mRNA (right panel) into the animal region of two dorsal blastomeres at the four-cell stage. **c**, **d** Embryos were classified into severe, mild, and normal groups on the basis of the extent of each defect. *P < 0.05 (with Bonferroni correction), Mann–Whitney *U*-test. **c** *N*, number of total right and left sides of embryos we evaluated. The right side and left side of each embryo were separately evaluated. **d** *N*, number of total eyes we evaluated. The right eye and left eye of each embryo were separately evaluated. **e**, **f** The body length of embryos at stage 40-41 was quantified (Uninjected, *n* = 59; HACE1 MO, *n* = 53; HACE1 MO + *myc-hace1* mRNA, *n* = 55). Values are expressed as the mean ± s.d. ***P < 0.001 (with Bonferroni correction), unpaired *t*-test

We next performed knockdown experiments in developing *Xenopus laevis* embryos by using an antisense morpholino oligonucleotide against *hace1* (HACE1 MO). We designed HACE1 MO to target the sequence containing the translation initiation AUG of *hace1* mRNA. To evaluate the specificity and efficiency of HACE1 MO, we performed immunoblotting analysis. HACE1 MO blocked the translation of C-terminally Myc-tagged *hace1* (*hace1-myc*) mRNA containing the HACE1 MO target sequence (Fig. 2a, left two lanes). We then injected HACE1 MO into the animal region of dorsal or ventral blastomeres at the 4-cell stage and observed the embryos at the tadpole stage (stage 40–41). Embryos that were dorsally injected with HACE1 MO displayed a number of defects, including a shortened body axis, a reduction of pigments, and a reduction of eye size (Fig. 2b, middle panel), whereas embryos that were ventrally injected with HACE1 MO exhibited no significant phenotype (data not shown). According to the extent of each defect, we classified embryos into three groups: severe, mild, and normal (Fig. 2c, d). Coinjection of HACE1 MO and N-terminally Myc-tagged *hace1* (*myc-hace1*) mRNA, which was MO-resistant because of the insertion of two Myc tags near the MO recognition site (Fig. 2a, middle and second right lanes), resulted in milder defects than the injection of HACE1 MO alone (Fig. 2b–f), indicating that the phenotypes observed in the HACE1 MO-injected embryos were primarily caused by Hace1 depletion. Thus, these results suggest that *hace1* has an important role in early *Xenopus laevis* embryonic development.

### Hace1 is required for convergent extension

**Fig. 3:**
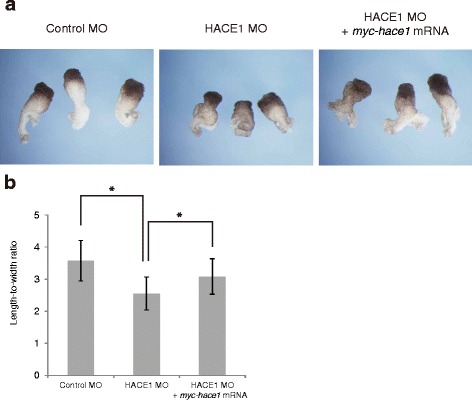
*hace1* knockdown inhibits convergent extension. **a** Keller sandwich explants were prepared from embryos injected with 30 ng of control MO or HACE1 MO into dorsal blastomeres at the four-cell stage. HACE1 MO (middle panel, *n* = 18), but not control MO (left panel, *n* = 18), caused a defect in elongation of explants. The defect in elongation caused by HACE1 MO was partly rescued by coinjection of 1.2 ng of *myc-hace1* mRNA (right panel, *n* = 18). **b** The length-to-width ratio of explants was quantified. Values are expressed as the mean ± s.d. *P < 0.05, unpaired *t*-test

In early *Xenopus laevis* development, dorsal mesodermal and ectodermal cells converge toward the midline and mediolaterally intercalate, and this process, called convergent extension, leads to elongation of the anterior-posterior body axis [10]. Therefore, it can be hypothesized that the shortened body axis observed in *hace1* knockdown embryos is caused by aberrant convergent extension. To test whether *hace1* regulates convergent extension, we used Keller sandwich explants, in which convergent extension can be reproduced in culture [11]. Explants from embryos injected with control MO (Fig. 3a, left panel) elongated normally, whereas those from embryos injected with HACE1 MO displayed little elongation (Fig. 3a, middle panel). The inhibition of elongation by HACE1 MO was rescued by coinjection of *myc-hace1* mRNA (Fig. 3a, right panel). To evaluate the elongation of explants, we measured the length-to-width ratio of explants. The length-to-width ratio was significantly reduced by HACE1 MO, and this reduction was significantly rescued by coinjection of *myc-hace1* mRNA (Fig. 3b). These results suggest that Hace1 regulates convergent extension.

### Hace1 depletion causes a delay in neural tube closure

**Fig. 4:**
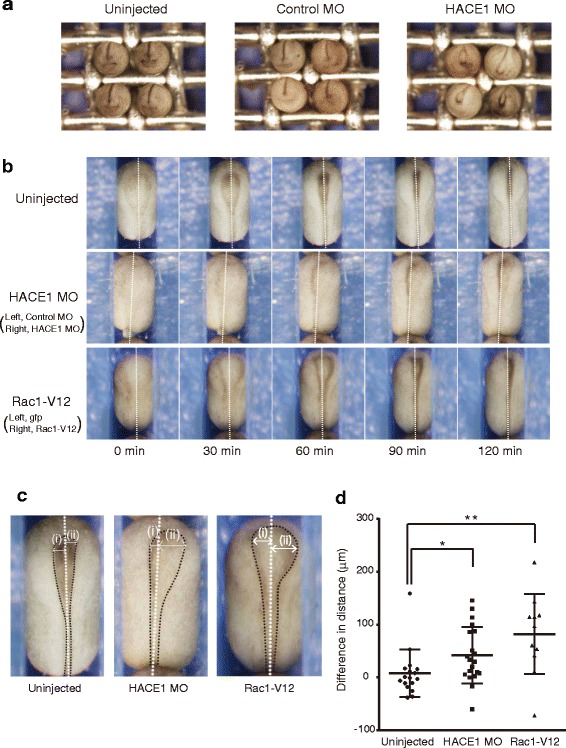
Knockdown of *hace1* and overexpression of *rac1-V12* cause a delay in neural tube closure. **a** Embryos were injected with the indicated MOs (30 ng) into the animal region of two dorsal blastomeres at the four-cell stage and the images were collected at stage 20. Anterior views with dorsal to the top. Representative results from two independent experiments are shown. **b** HACE1 MO (15 ng) was injected into the animal region of the right dorsal blastomere, and control MO (15 ng) was subsequently injected into that of the left dorsal blastomere. *rac1-V12* (constitutively active Rac1) mRNA (10 pg) and *gfp* mRNA (90 pg) were injected into the animal region of the right dorsal blastomere, and then *gfp* mRNA alone was injected into the animal region of the left dorsal blastomere. Embryos at stage 13 were placed on a culture dish, and images were collected every 5 min until embryos completed neural tube closure. Still frames from a time-lapse movie show embryos from stage 14 (0 min) to stage 18 (120 min). Dorsal views with anterior to the top. Dotted lines indicate the midline. Transient posterior protrusion (e.g., middle left) was sometimes observed because embryos were firmly embedded in the agarose groove and thus were constantly subjected to the lateral-to-medial force. **c** A magnified view of embryos in b (60 min). Black dotted lines indicate the inner edges of neural folds. **d** The maximum distance between the dorsal midline and the left (i) or right (ii) inner edge of the neural fold was measured (shown as white double-headed arrows in c). The differences ((ii) minus (i)) were then calculated and plotted (Uninjected, *n* = 17; HACE1 MO, *n* = 20; *Rac1-V12*, n=11). Long central bar marks mean and upper and lower bars mark s.d. *P < 0.05, **P<0.01, Mann–Whitney *U*-test


Because HACE1 MO inhibited convergent extension in Keller sandwich explants, we examined the effect of HACE1 MO on gastrulation and neural tube closure, which are two major morphological events involving convergent extension, in whole embryos. An injection of 30 ng of HACE1 MO did not lead to an apparent defect in gastrulation (data not shown), but it led to a delay in neural tube closure at the neurula stage (Fig. 4a). To examine this phenotype in detail, we performed time-lapse imaging of neurulating embryos. At the 4-cell stage, HACE1 MO was injected into the right dorsal blastomere, and control MO was injected into the left dorsal blastomere (Fig. 4b–d, indicated as HACE1 MO). As a control, uninjected embryos were also observed (Fig. 4b–d, uninjected). Embryos at stage 13 were placed on a culture dish, and images were collected every 5 minutes until neural tube closure was completed (Fig. 4b–d and see also Additional file 1: Movie S1 and Additional file 2: Movie S2; uninjected embryos and MO-injected embryos, respectively). The neural plate was likely to be formed in the HACE1 MO-injected right side (Fig. 4b, 0 min). However, the elevation of the neural fold was much less apparent in the HACE1 MO-injected side than in the control MO-injected side (Fig. 4b, the middle panel at 60 min). Although the neural fold became visible at 30 to 60 min in the HACE1 MO-injected right side, the ridge of the neural fold in the right side was much less prominent than that in the control MO-injected left side (Fig. 4b, 120 min). We measured the maximum distance between the dorsal midline and the inner edge of the neural folds. In uninjected embryos, the distance in the right side was roughly equal to that in the left side (Fig. 4c, d). However, the distance in the HACE1 MO-injected right side was markedly longer than that in the control MO-injected left side (Fig. 4c, d), thus indicating that knockdown of *hace1* led to a significant delay in neural tube closure. These results suggest that *hace1* is required for normal neural tube closure.

**Additional file 1: Movie S1.** Neural tube closure of an uninjected embryo.


**Additional file 2: Movie S2.** Neural tube closure of an embryo injected with HACE1 MO.

### Phenotypes of *hace1* morphants are specific for loss of *hace1*

**Fig. 5:**
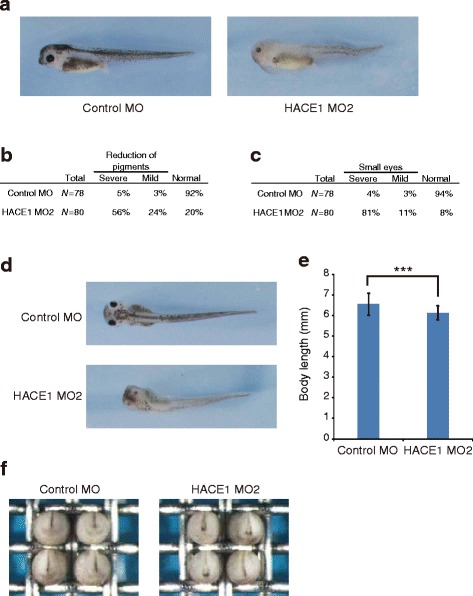
Knockdown of *hace1* by HACE1 MO2 leads to a phenotype similar to that caused by HACE1 MO treatment. **a** Embryos were injected with 50 ng of Control MO (left panel) or 50 ng of HACE1 MO2 (right panel) into dorsal blastomeres at the four-cell stage and fixed at stage 38/39. Representative results from two independent experiments are shown (Control MO, *n* = 39; HACE1 MO2, *n* = 40) (**b**, **c**) Embryos were classified into severe, mild, and normal groups on the basis of the extent of each defect. **b** *N*, number of total right and left sides of embryos we evaluated. The right side and left side of each embryo were separately evaluated. **c** *N*, number of total eyes we evaluated. The right eye and left eye of each embryo were separately evaluated. **d**, **e** The body length of embryos at stage 38/39 was quantified (Control MO, *n* = 39; HACE1 MO2, *n* = 40). Values are expressed as the mean ± s.d. ***P < 0.001, unpaired *t*-test. **f** Embryos were injected with 50 ng of Control MO (left panel) or 50 ng of HACE1 MO2 (right panel) into dorsal blastomeres at the four-cell stage and the images were collected at stage 20 (Control MO, *n* = 40; HACE1 MO2, *n* = 40). Anterior views with dorsal to the top

Knockdown by antisense morpholino oligonucleotides may be accompanied by off-target effects [12]. Although we performed rescue experiments (Fig. 2b–f), we further evaluated the specificity of *hace1* knockdown. We designed HACE1 MO2 as a second non-overlapping morpholino oligo for *hace1*. Whereas HACE1 MO targets a region from −1 to +24 from the translational start site, HACE1 MO2 targets a region from −79 to −55 from the translational start site. Embryos dorsally injected with HACE1 MO2 displayed a shortened body axis, a reduction of pigments, and a reduction of eye size (Fig. 5a–e). We next examined the effect of HACE1 MO2 at an early developmental stage. Similarly to the results from the injection of HACE1 MO, the injection of HACE1 MO2 did not lead to an apparent defect in gastrulation (data not shown), but it did lead to a delay in neural tube closure at the neurula stage (Fig. 5f). Thus, knockdown of *hace1* by HACE1 MO2 led to essentially the same phenotype as that by HACE1 MO. These results further confirmed the specificity of morpholino-mediated knockdown of *hace1*.

### Developmental defects in *hace1* morphants are largely caused by an excess of active Rac1

**Fig. 6:**
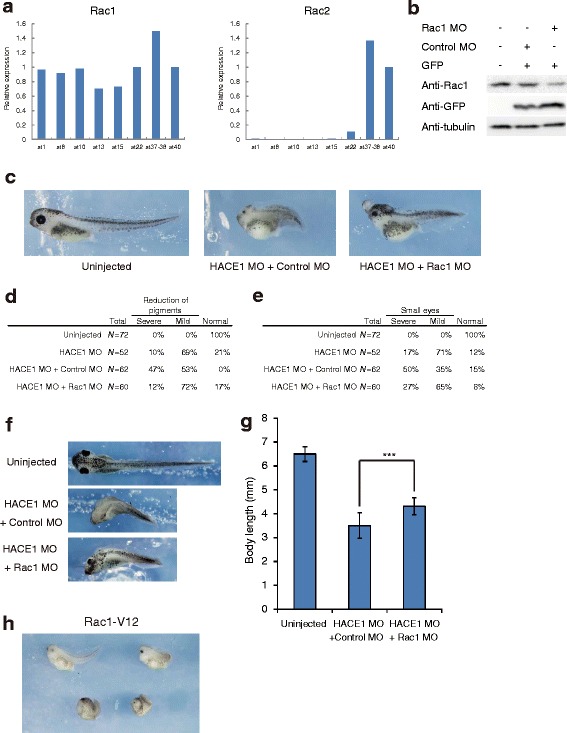
Defects in *hace1* morphants are partly rescued by coinjection of Rac1 MO. **a** Temporal expression patterns of *Xenopus laevis rac1* and *rac2*. The expression levels of *rac1* and *rac2* were analyzed by qRT-PCR. Expression levels were normalized to those of *Xenopus laevis ornithine decarboxylase 1* (*odc1*). **b** Immunoblot analysis shows that Rac1 MO inhibited translation of endogenous Rac1. *Xenopus laevis* embryos were injected with the indicated MOs (70 ng in total) and *gfp* mRNA (0.2 ng). α-tubulin was a loading control. **c** Coinjection of Rac1 MO (50 ng) and HACE1 MO (30 ng) led to milder developmental defects than did coinjection of control MO (50 ng) and HACE1 MO (30 ng). Anterior is to the left, and dorsal is to the top. **d**, **e** Embryos were classified into severe, mild, and normal groups on the basis of the extent of each defect. **f**, **g** The body length of embryos at stage 40-41 was quantified. Dorsal views of embryos are shown with anterior to the left. Values are expressed as the mean ± s.d. ***P < 0.001, unpaired *t*-test **h** Embryos injected with 30 pg of *Rac1-V12* (constitutively active Rac1) mRNA and 170 pg of *gfp* mRNA displayed phenotypes similar to those of embryos injected with HACE1 MO (*n* = 38). Anterior is to the left, and dorsal is to the top

HACE1 ubiquitinates GTP-bound RAC1 and causes its degradation [3]. Thus, it is possible that the phenotype of *hace1* morphants is caused by an increase in active Rac1. To investigate the relationship between *hace1* and *rac*, we first attempted to analyze the expression pattern of the *rac* family genes: *rac1*, *rac2* and *rac3*. It has previously been shown that *Xenopus laevis rac1* is expressed in neural tissues at the neurula and tailbud stages [13]. Our qRT-PCR analysis showed that *rac1* is expressed throughout early development, and *rac2* expression starts from the early tailbud stage (Fig. 6a). The full-length coding sequence of *Xenopus laevis rac3* has not been deposited in GenBank, and we were unable to find *Xenopus laevis* EST sequences corresponding to *rac3*. In addition, we were unable to amplify any DNA fragments from *Xenopus laevis* embryos in our RT-PCR experiments using primers that were designed on the basis of the *rac3* sequence from the closely related species *Xenopus* (*Silurana*) *tropicalis*, thus suggesting that *rac3* may be absent or weakly expressed in *Xenopus laevis* embryos. These results indicate that *rac1* is predominantly expressed among *rac* family members at the neurula stage. Thus, it is possible that *hace1* targets *rac1* in *Xenopus laevis* neurulation.

We next asked whether the defects observed in *hace1* morphants could be rescued by reducing Rac1 activity. We used Rac1 MO to reduce the total Rac1 protein level. Immunoblotting analysis confirmed that Rac1 MO reduced the protein level of endogenous Rac1 by more than half (Fig. 6b). We injected HACE1 MO with control MO or Rac1 MO into embryos and fixed them at the tadpole stage (stage 40–41). Embryos coinjected with HACE1 MO and Rac1 MO displayed milder defects in eyes and pigments than did embryos coinjected with HACE1 MO and control MO, although these double MO-injected embryos displayed more severe phenotypes than embryos injected with HACE1 MO alone, possibly because of overdosing of MOs (Fig. 6c–e). In addition, embryos coinjected with HACE1 MO and Rac1 MO had a longer body length than did embryos coinjected with HACE1 MO and control MO (Fig. 6f, g). These results suggest that the defects in *hace1* morphants are at least partly caused by an excess of active Rac1.

To further test this possibility, we expressed *rac1-V12*, a constitutively active mutant of *rac1*, in the presumptive neural plate to examine the effect of excessive active Rac1 on neurulation. Nearly half of the embryos injected with *rac1-V12* mRNA displayed a phenotype similar to that of embryos injected with HACE1 MO, such as a shortened body axis (Fig. 6h, compare with Fig. 2b, middle), thus supporting our idea that developmental defects in *hace1* knockdown embryos are at least partly due to excessive active Rac1. Because it has previously been reported that exogenous expression of *rac1-V12* in the mesoderm (1 or 2 ng of *rac1-V12* mRNA into the marginal zone of embryos) results in gastrulation defects [14], we carefully observed the effect of *rac1-V12* on gastrulation and showed that injection of *rac1-V12* mRNA in our experimental condition (30 pg of mRNA was injected into the animal region of two dorsal blastomeres at 4-cell stage) did not affect gastrulation (data not shown).

### Excess of active Rac1 causes a delay in neural tube closure

To further characterize the phenotype of the embryos injected with *rac1-V12* mRNA, we performed time-lapse imaging and quantified the delay in neural tube closure. Embryos in which *rac1-V12* mRNA was injected into the right side showed a significant delay in neural tube closure in the injected side, as compared with uninjected embryos (Fig. 4b–d and see also Additional file 1: Movie S1 and Additional file 3: Movie S3; uninjected embryos and *rac1-V12* mRNA-injected embryos, respectively). These results support our hypothesis that *hace1* regulates *Xenopus laevis* neural tube closure through Rac1 degradation.


**Additional file 3: Movie S3.** Neural tube closure of an embryo injected with Rac1-V12 mRNA.

### *hace1* has a role in regulating differentiation to neural ectoderm

**Fig. 7:**
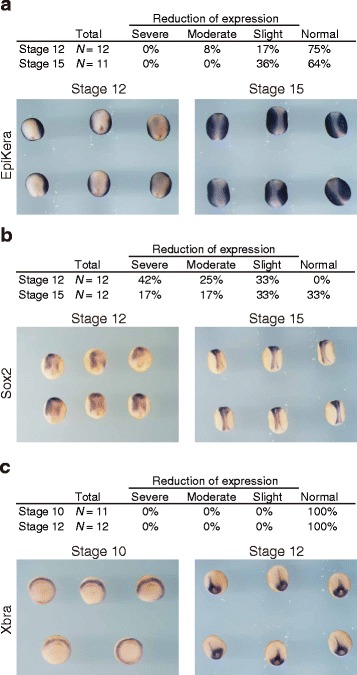
*hace1* has a role in regulating differentiation into neural ectoderm. **a**–**c** HACE1 MO (15 ng) was injected into the animal region of the right dorsal blastomere, and control MO (15 ng) was injected into that of the left dorsal blastomere at the 4-cell stage. Embryos were fixed at the stages indicated, and expression of *epidermal keratin*, an epidermal ectoderm marker (**a**), *sox2*, a neural ectoderm marker (**b**), and *Xbra*, a mesodermal marker (**c**), were analyzed by in situ hybridization. Representative images from two independent experiments are shown (lower panels)

Although the results shown above suggest that convergent extension is a major process affected by *hace1* depletion, it is possible that *hace1* regulates cell fate specification, such as differentiation into the epidermal ectoderm, neural ectoderm, and mesoderm. To test this possibility, we analyzed the expression of marker genes of cell fate specification in *hace1* depleted embryos. HACE1 MO was injected into the right side, and control MO was injected into the left side; the expression of marker genes was detected by in situ hybridization. Whereas the expression of the epidermal ectoderm marker *epidermal keratin* and the mesoderm marker *Xbra* were unaffected on the HACE1 MO-injected side (Fig. 7a, c), *hace1* depletion resulted in a moderate or slight decrease in the expression of the neural ectoderm marker *sox2* at stage 12 (late gastrula) or stage 15 (early neurula), respectively (Fig. 7b). Additionally, the distribution pattern of all of these marker genes was essentially unaffected in HACE1 MO-injected side. According to the extent of reduction, we classified embryos into four groups: severe, moderate, slight, and normal (Fig. 7a–c). These results suggested that *hace1* is likely to be involved in differentiation to the neural ectoderm. Although it is difficult to determine whether this level of decrease in Sox2 affects the subsequent neural tube closure event, *hace1* might regulate neural tube closure through controlling differentiation to the neural ectoderm.

## Discussion

In this study, we investigated the role of the E3 ubiquitin ligase Hace1 in early embryonic development. We showed that *Xenopus laevis hace1* is expressed in neural tissues and the kidney. High-throughput in situ hybridization in mouse embryos has revealed that *Hace1* is expressed in neural tissues in mice at embryonic day 11.5 (E11.5), E15.5, and postnatal day 7 [15]. In humans, *HACE1* is expressed in adult tissues including the heart, brain, and kidney [1]. These data suggest that HACE1 expression in neural tissues is common in vertebrates.

Hace1 depletion in *Xenopus laevis* embryos led to a shortened body axis. The process of body axis elongation is known to involve convergent extension [10, 16, 17]. Our analysis with Keller sandwich explants showed that knockdown of *hace1* inhibited convergent extension. These results suggest that *Xenopus laevis hace1* may control body axis elongation through regulation of convergent extension. Convergent extension occurs in both dorsal mesodermal and posterior neural tissues. Whereas convergent extension in the dorsal mesoderm mainly regulates gastrulation, convergent extension in the neural ectoderm mainly regulates neurulation. It has been shown using transplantation experiments that inhibition of convergent extension in only the neural ectoderm leads to a shortened body axis [18]. Because knockdown of *hace1* did not affect gastrulation, inhibition of convergent extension in the neural ectoderm by *hace1* depletion might lead to a shortened body axis.

Notably, *Hace1*^−/−^ mice do not display any developmental defects [2], whereas knockdown of *hace1* in *Xenopus laevis* led to severe developmental defects. This discrepancy might be explained by the possibility that other proteins with roles redundant with that of HACE1 may compensate for HACE1 function during embryonic development of *Hace1*^−/−^ mice. For instance, the activity of Rac1, one of the targets of HACE1, is negatively regulated by GAP and other E3 ubiquitin ligases targeting Rac1, such as IAPs [19–21]. Ectodermal tissues in mouse embryos might express these proteins to provide robustness to loss of Hace1 protein. Most recently, it has been reported that knockdown of *hace1* using a splice-blocking MO causes ROS production in zebrafish [5]. Although this report has not described the effects of the MO on zebrafish embryonic development, the gross morphology of embryos injected with the MO is apparently normal. Whereas a splice-blocking morpholino inhibits only zygotic expression but not maternal expression, a translation blocking morpholino, which was used in our study, inhibits both maternal expression and zygotic expression. This might explain the difference between our results and those from the previous study in zebrafish.

*hace1* depletion in *Xenopus laevis* embryos led to diverse developmental defects, including a shortened body axis and the inhibition of eye and pigment formation. These defects were at least partly rescued by *rac1* inhibition. In addition, we showed that overexpression of active Rac1 in the presumptive neural plate led to a delay in neural tube closure and a number of subsequent developmental defects, which are similar to *hace1* morphants. The migration of neural crest cells, which give rise to pigment cells, is disturbed by constitutively active or dominant negative forms of Rac1 [22]. Therefore, the inhibition of pigment formation may result from impaired neural crest migration. Additionally, conditional knockout of *Rac1* in the surface ectoderm results in a failure of neural tube closure in mice [23]. Thus, fine tuning of Rac1 activity may be important for neural tube closure. However, the detailed molecular mechanism by which excessive active Rac1 affects neural tube closure is unclear. Rac1 regulates a variety of biological processes, including cytoskeleton remodeling and PCP signaling regulation [24, 25]. PCP signaling plays an important role in neural tube closure [26], and there may be crosstalk between RhoA and Rac1 signaling in the regulation of actin cytoskeleton [27]. It would be interesting to investigate the presence of the crosstalk and, if it is present, to clarify the regulation mechanism of the PCP pathway through RhoA and Rac1 signaling during neural tube closure.

## Conclusions

In summary, our study show that the E3 ubiquitin ligase Hace1 plays an important role in early developmental processes in *Xenopus laevis*. Detailed analyses of downstream targets of Hace1 should be performed in future studies.

## Methods

### Molecular cloning and plasmid construction

Primers were designed on the basis of the *Xenopus laevis hace1* sequence (GenBank accession NM_001093608) as follows: F, 5′-GGTAGATCTATGGAGAGAGCAATGGAGCAACTC-3′; R, 5′-GGTAGATCTTTATGCCATTGTGTAGCCGTAGCT-3′, and the *Xenopus laevis rac1* sequence (GenBank accession NM_001095863) as follows: F, 5′- CGGAAGATCTGTAGGGAGAGCAAAGAAGAGGGAGGGAG-3′; R, 5′- TCCGGAATTCAGGGACAGAAGAAAAGATGGCATGTGGG-3′. PCR was performed with complementary DNAs derived from embryos at stage 2 for *hace1* and stage 13 for *rac1*. The entire amplified coding sequences were cloned into the expression vector pCS2+. A constitutively active form of *Xenopus rac1* was constructed by replacing glycine 12 with valine by site-directed mutagenesis.

### Whole mount in situ hybridization

Whole-mount in situ hybridization was performed on albino *Xenopus laevis* embryos according to a standard protocol [28] using a robot (InSituPro, Intavis). The digoxigenin-labeled probes were synthesized from *hace1*-pCS2+.

### Embryo manipulation

*Xenopus laevis* embryos were obtained by in vitro fertilization and cultured in 0.1 × MBS at 15 or 22 °C. Embryos were staged according to Nieuwkoop and Faber [29]. Antisense morpholino oligonucleotides (MOs) and mRNAs were injected into the animal region of one or two dorsal blastomeres at the four-cell stage in 4 % Ficoll in 0.1× MBS. In vitro synthesis of capped mRNA was performed using the mMESSAGE mMACHINE (Ambion). MOs were purchased from Gene Tools. The MO sequences were as follows: HACE1 MO, 5′- GAGTTGCTCCATTGCTCTCTCCATC-3′; HACE1 MO2, 5′- AGAGGCTCAGCAGTTCCTAAGCAGT-3′; Rac1 MO, 5′-CCACACATTTAATGGCCTGCATGGC-3′; and a standard control oligo (Control MO), 5′-CCTCTTACCTCAGTTACAATTTATA-3′. The animals were bred and handled with care, according to a published laboratory manual [28]. *Xenopus laevis* experiments complied with the Regulation on Animal Experimentation at Kyoto University and were approved by the Animal Experimentation Committee of Kyoto University.

### Immunoblotting

Embryos were lysed in a buffer consisting of 20 mM Tris–HCl, pH 7.5, 150 mM NaCl, 1.5 mM MgCl_2_, 2 mM EGTA, 25 mM β-glycerophosphate, 10 mM sodium pyrophosphate, 1 % Nonidet P-40, 10 mM NaF, 1 mM vanadate, 1 mM DTT, and 1× Protein Inhibitor Cocktail (Sigma). Extracts were then centrifuged, and supernatants were collected. Anti-Myc (A14, Santa Cruz), anti-GFP (JL8, Clontech), anti-Rac1 (23A8, Millipore) or anti-α-tubulin (DM1A, Sigma) antibodies were used as primary antibodies, and anti-mouse IgG HRP-conjugated (1:10,000; GE healthcare) and anti-rabbit IgG HRP-conjugated (1:10,000; GE healthcare) were used as secondary antibodies.

### Quantification of body length

Embryos were fixed in MEMFA [100 mM MOPS (pH 7.4), 2 mM EGTA, 1 mM MgSO_4_, 3.7 % formaldehyde] at stage 40–41. Embryos were placed on agarose gel, and images were collected. The body length was measured along the dorsal midline using the AxioVision (Zeiss) measure tool.

### Keller sandwich explant analysis

Keller sandwich explant experiments were performed as previously described [30]. MOs and mRNA were injected into the animal region of two dorsal blastomeres at the four-cell stage. Injected embryos were grown to stage 10.5 for preparation of Keller sandwich explants. Explants were cultured in Sater’s modified blastocoel buffer containing 0.1 % BSA until uninjected embryos reached stage 19. The length and width of explants were measured using cellSens software (Olympus).

### Time-lapse analysis of neural tube closure

Embryos injected with MOs or mRNAs were grown to stage 13 and placed dorsal side up in a 60 mm culture dish. This dish contained 2 % agarose with a groove made by an acrylic comb. Images were collected with an Olympus SZX16 microscope, and time-lapse movies were obtained using cellSens software. For quantitative analysis of the delay in neural tube closure, the maximum distance between the dorsal midline and the left (i) or right (ii) inner edge of the neural fold was measured at the time point 60 min after start of time-lapse imaging, as shown in Fig. 4c. At the time point 60 min after start of time-lapse imaging, embryos reached stage 17. The inner edge of the neural fold was easily visible, owing to pigmentation.

### Quantitative RT-PCR analysis

Total RNA isolation and cDNA synthesis were performed as previously described [31]. The gene expression levels were normalized to those of *odc1* (ornithine decarboxylase 1). The sequences of primers used were as follows: *Xenopus laevis rac1* (NM_001095863) (F, CATGCACATGTCAAGCCAGTTC; R, ATGGCAAGTCCCTGAGGATAGG); *Xenopus laevis rac2* (NM_001092288) (F, ACCAGTAAACTTGGGCTTGTGG; R, CTCATAAGATGCCGGACTCACC); *Xenopus laevis odc1* (NM_001086698) (F, TGAAAGTGGCAAGGAATCACCC; R, GATACGATCCAGCCCATCACAC).

## Abbreviations

GAP: GTPase-activating protein
HACE1: HECT domain and ankyrin repeat containing E3 ubiquitin protein ligase 1
IAP: Inhibitors of apoptosis protein
MO: Morpholino oligonucleotides
ROS: Reactive oxygen species
